# Supplementary material for: Are carbon emissions trading and green financial instruments synergistic? -Comprehensive quantitative research based on content analysis
Source: PLoS One. 2024 Mar 7;19(3):e0298601. doi: 10.1371/journal.pone.0298601 (PMC10919601; doi:10.1371/journal.pone.0298601)
Supplement: S1 Data — (ZIP) [file pone.0298601.s001.zip › Data/地级市Z_F工作报告.zip!/┴╔─■/╡ñ╢1⁄2╩╨/└┤╫╘╣1⁄2╓┌║┼í╛┬φ┐╦╩2╛▌═°í┐_2010.docx]

各位代表：

现在，我代表市人民政府向大会报告工作，请予审议，并请市政协委员和其他列席人员提出意见和建议。

一、2009年政府工作的回顾

2009年，在省委、省政府和丹东市委的正确领导下，面对历史罕见的金融危机、前所未有的严峻挑战、更加繁重的发展任务，市政府组织和带领全市人民，紧紧抓住国家扩大内需和辽宁沿海经济带上升为国家发展战略的重大机遇，提升应对挑战、化解矛盾的精神状态，坚定加快发展、大干快上的信心勇气，集聚乘势而上、攻坚克难的发展合力，保持了经济社会发展的向好势头，较好完成了市十四届人大二次会议确定的各项任务。

——经济又好又快发展，主要指标实现较大突破。预计全地区生产总值完成655亿元，比上年增长16.2%；规模以上工业增加值191.8亿元，增长23%；财政一般预算收入实际完成50.1亿元，增长33.4%；预计全社会固定资产投资完成525亿元，增长50.2%；外贸出口14.2亿美元，增长2.5%；实际利用外商直接投资5亿美元，增长63.4%；引进内资241亿元，增长101%；社会消费品零售总额234亿元，增长18.2%；城镇居民人均可支配收入12826元，增长10.2%；农民人均纯收入7370元，增长11.2%；万元GDP能耗1.49吨标准煤，下降4.6%。

——项目建设成效显著，数量增加质量不断提升。去年是我市推进实施项目数量最多、投资规模最大的一年，在建项目的数量、质量、单体投资规模均创历史最好水平。全年在建和开工千万元以上项目578个，其中亿元以上项目98个，项目拉动税收增收7.8亿元，占税收增量比重超过80%。60个重大推进项目开工45个，竣工或部分竣工12个。举世关注的新建鸭绿江公路大桥项目正式启动，完成选址；沈丹铁路客运专线、丹大快速铁路即将开工建设；本钢丹东不锈钢冷轧薄板项目桩基工程完工，是近年来引进的少有的国企投资大项目；投资估算500亿元的丹东核电项目正在积极做前期工作。续建中的蒲石河抽水蓄能电站、三湾水利、鸭绿江西水道等项目进展顺利。东北东部铁路，丹通、丹海高速公路，金山热电，机场改扩建，翁泉沟硼铁综合利用等项目实现开工建设并保证了年度投资进度。500千伏输变电、丰能风电环锻件、凤辉硼业含硼特钢等项目已竣工投产或试生产。丹东港打通西南航道，完成了2个5万吨级粮食泊位等工程建设。全社会港口吞吐量达到4350万吨，增长25.3%。争取中央新增投资项目147个，争取国家资金12.6亿元。

——新区建设展露形象，产业园区打造“丹东速度”。新区建设呈现突飞猛进的态势，创造出一年即见形象的奇迹。新开工项目195个，总投资236亿元。基础设施配套更加齐备，投入资金25亿元，新整理土地9平方公里，形成了“六纵六横”的主干路网，完成了配套基础设施及绿化、亮化工程建设，项目入驻和城市发展的基础已经夯实；城市功能快速形成，二中新校区、国门公园、市民公园、灯塔山公园等全部投入使用，文化中心、体育中心、九年制义务教育学校、市民服务中心等正在加紧建设，人口和产业聚集的基本条件已经形成；商贸项目进展顺利，新开工新世纪佳园等9个商贸地产项目，城市中央商务区的框架已经搭起；支柱产业迅速集聚，仪器仪表产业基地共建成标准厂房50万平方米，研发检测中心、物流配送中心正在建设，辽宁仪器仪表学院主体工程完工，有120个企业完成注册，172个项目签订投资协议。产业园区有纳诺工业园、东大电机等186个工业项目开工建设，总投资达142亿元，其中曙光乘用车、五一八镦锻曲轴等46个项目竣工投产或试生产。环保产业园完成土地整理1平方公里，已入驻企业27户，其中12户开工建设。

——财政收入稳步增长，金融支持发挥重大作用。财政一般预算收入首次突破50亿元大关。其中，各项税收完成35.3亿元，增长37.8%；财政非税收入完成14亿元，占财政一般预算收入的28%。财政一般预算支出突破100亿元，用于支持经济社会发展、民生、农业、教育等重点支出比上年增加26.2亿元，占财政支出增量近90%。预计全年存款增长29.4%；贷款增长50%，其中新增贷款是上年的6倍多。城投公司新增资产105亿元，实现银行融资31.3亿元。获批2家村镇银行正在筹建，获批14家小额贷款公司已开业4家。欣泰电气实现创业板上市申报。

——工业经济企稳向好，措施得力促进逆势上扬。预计主营业务收入710亿元，增长32%；实现税金29.2亿元，增长20%；新增规模以上企业70户，其中超亿元企业20户。工业固定资产投资预计完成240亿元，增长60%，占全社会固定资产投资比重达45.3%。新开工千万元以上项目120个，其中亿元以上项目40个，是历史上新开工亿元以上项目最多的一年。启动停产企业197户，占停产企业总数的90%。为重点用电企业解难题、给政策，在下半年即实现用电正增长。制定并实施了10条优惠政策，促进企业扩大销售，预计全年规模以上工业产品销售率达到98.5%，同比增加0.7个百分点。高新技术产业增加值预计增长33.5%，新建企业技术研发中心23个。试成新产品480项，投产240项。完成海外并购科技型项目2个。化纤公司破产重整工作取得突破性进展，多年遗留的改革难题初步得到解决。

——“三农”工作不断推进，县域经济实现提速升位。第一产业增加值实现两位数增长，粮食产量88.3万吨，蓝莓、草莓、柞蚕继续保持全国产量第一的地位。新增规模以上农产品加工企业30家。农产品出口创汇近4亿美元，建设出口安全农产品示范基地140万亩，已经获国家批准的“三品”认证60个。新增设施农业面积2.5万亩，畜牧小区完成203个。伏季休渔是历年来工作力度最大、管控效果最好的一年。鸭绿江流域增殖放流共投放各类鱼苗近千万尾。推介农业新品种、新技术50项，培训农民3.7万人。投入资金3亿多元，实施了农田水利系列工程，是近年来投入资金和工程项目最多的一年。投资2亿元开展土地开发整理，新增耕地512公顷。发放各类惠农补贴资金2.67亿元。林权制度主体改革全面完成。县域经济快速发展，东港市、凤城市被省政府确定为三年冲击全国百强县，东港市有望进入2009年度百强县。五个边境县（市）区全部纳入“兴边富民行动”国家重点县扶持行列。

——商贸服务繁荣活跃，旅游产业凸显丹东亮点。沃尔玛超市落户丹东，“北国之春”温泉度假村、天桥沟旅游综合开发项目已经开工建设，一批商贸项目加快推进。“新网工程”、“家电下乡”等“促消费、保增长”活动走在全省前列。商品房促销等措施成效明显，商品房销售增长97%。促进城区经济加快发展的各项保障措施全面落实。虎山长城确定为万里长城东端起点。鸭绿江国际旅游节上升为国家级节庆活动，办出了特色和水平，成功举办了辽宁台湾周活动。精心打造鸭绿江旅游品牌和“一江四线”精品线路，虎山景区综合改造项目动迁和规划工作基本结束。正式开通了赴朝边境旅游异地办证试点业务。旅游宣传促销覆盖到长三角、珠三角等地区。旅游业各项指标有大幅度增长。

——对外开放再创佳绩，主题招商引领投资热潮。招商引资实现历史性突破，引进内资、外资增幅均位居全省前列。以仪器仪表、环保产业和矿产资源开发等为重点的十大产业集群初具规模，共引进项目75个，总投资额124亿元。再生资源园区、硼化工园区等重点园区初现形象，全市各园区共建设标准厂房100万平方米。外贸出口在全省普遍负增长的形势下保持了正增长。高水平规划了沿江沿海“四大组团”，招商和建设工作全面启动。东北东部12+1经济合作工作机制初步建立，东北东部经济带建设取得实质性进展。

——惠民实事件件落实，民生工程受到群众好评。投入6.2亿元，完成了为民15件实事各项进度。新建农村饮水安全工程受益农民11.3万人。五龙背自来水供水项目的建设使1.3万人饮用水安全得到了保证。完成农村公路建设400公里，提前一年实现村村通油路目标。三条快速公路干线基本完工。新建5座污水处理厂并全部竣工。实施城建项目25个，改造大修29条城市道路，开工建设了两条城市南北干线，80个旧小区改造全面完成，市容市貌得到整治。丹东东港同城化各专项规划和共建共享项目顺利推进，同城连接带城际道路升级改造亮化等一批重点基础设施初具规模，公共服务项目相继立项或开工建设，金融、教育、卫生、社保、就业等方面已经局部或全面实现了同城化。建设完成2万平方米、439套廉租房，对农村2050户困难户危房进行了改造。新增就业6万人，82户零就业家庭中110人就业，兜底安置困难大学生96人，登记失业率控制在4.5%以内。省下达各项社会保险扩面指标全面完成。城乡低保户、五保户和重点优抚对象普遍提高了救助优待标准。

——各项事业全面进步，社会稳定人民安居乐业。义务教育均衡发展步伐加快，素质教育不断加强，完成了32万平方米中小学校舍改扩建任务，新丹东二中成为我市规模最大、设施最好的省级示范高中。13个引进海外研发团队项目获省政府批准。农村公共文化建设稳步推进，文化中心等一批体育文化基础设施开始建设。新农合人均筹资标准达到100元。在全省第一个实现妇幼卫生网络系统管理。市中医院等重点医疗机构建设工程顺利竣工。计划生育特别扶助制度全面落实。十一届全运会丹东籍健儿勇创佳绩。高质量完成元宝山广播电视观光塔建设和有线电视“村村通”工程。“政风行风热线”和“马上就办”栏目成为政府服务民生的有效途径。信访和维稳工作扎实有效，各类刑事犯罪活动和非法宗教活动得到遏制，社会治安防控体系进一步完善。国庆安保工作圆满完成。法律保障能力逐步提升。应急管理工作成效显著。安全生产总体保持平稳。争创省双拥模范城中期评选工作得到省检查组的高度评价。顺利完成了政府机构改革，公务员队伍管理水平得到提高，行政效能和勤政廉政建设不断加强。

统计、外事、侨务、台务、民族、宗教、残疾人、保密、档案、接待、人防、气象、地方志、国资监管、防震减灾等工作也取得了新成绩。

各位代表，过去的一年，我们迎来了辽宁沿海经济带上升为国家战略的历史机遇，见证了新中国成立60周年的辉煌时刻，抵御了国际金融危机的严重冲击。全市上下在趋利避害中抢抓机遇、经受挑战、迎难而上，取得了来之不易的成绩，这是省委、省政府和市委正确领导的结果，是市人大依法监督、市政协民主监督并给予大力支持的结果，是我们发扬“白加黑”、“五加二”的工作作风和落实落实再落实的工作精神，顽强拼搏，共同奋斗的结果。在此，我谨代表市政府，向各个领域和岗位上辛勤劳动、做出贡献的全市人民，向解放军和武警驻丹部队，向人大代表、政协委员、各民主党派、工商联、无党派人士和各界朋友，向中省直驻丹单位，表示衷心的感谢并致以崇高的敬意！

在看到成绩的同时，也必须清醒地认识到，我市经济社会发展中仍然存在一些不容忽视的问题。一是经济结构不尽合理。经济总量在全省排名靠后；规模工业增加值占GDP比重不足30%，支撑地区经济发展的能力较弱。二是工业大项目依然不多。至今我市还没有一个单体投资超百亿元的工业项目；项目进展不够平衡，政府投资项目开工率较高,进展快,而民间投资项目开工少,进展相对迟缓；基础设施项目开工多,规模大,而工业大项目落地及开工仍然较少；集群类项目不多,特别是具备龙头带动作用项目匮乏。三是城区经济总量在全省排名靠后。城区原有工业企业或倒闭或大部分已搬迁出市区，接替主导产业还没有形成；各城区经济结构大同小异，产业趋同现象比较明显，城区之间经济互补性差，重复建设现象突出。四是外贸出口形势依然严峻。缺少出口大企业和拳头产品，出口产品结构不尽合理，高新技术和精深加工产品少，产品附加值低，市场竞争力不强。五是财政压力仍然很大。税收收入基础不够稳固，缺乏可持续性支柱财源；市本级财政一般预算收入占财政一般预算收入比重仍然偏低；刚性支出连年快速增长，市本级财政历史包袱沉重，财政支出压力较大的矛盾仍然没有解决。六是城市管理水平不高。多年形成的占道经营、私搭乱建、牌匾乱挂等问题没有彻底得到根治，环境卫生还存在许多问题；由于资金不足，城市公用设施建设欠帐较多，设施老化，影响群众生活。七是软环境建设还需要进一步加强。少数干部作风不实，乱作为、不作为、慢作为等问题影响了投资软环境建设。这些问题，需要在今后的工作中切实加以解决。

二、2010年经济和社会发展的主要任务

今年是实施“十一五”规划的收官之年，也是我市抢抓辽宁沿海经济带上升为国家战略的有利机遇，站在新起点，实现新跨越的关键之年。当前，国际金融危机的影响仍然存在，国内经济回升内在动力仍然不足，但我们应该在困难中看到机遇，在挑战中坚定信心。信心来自于对机遇的有效把握，要看到辽宁沿海经济带上升为国家发展战略，新的鸭绿江公路大桥即将建设，丹东从未像现在这样令世人瞩目；信心来自于对形势的正确判断，要看到中央、省里已经出台一系列扩大内需、促进增长的政策措施，丹东从未像现在这样受到国家和省委、省政府的高度重视；信心来自于对现状的全面认识，要看到近年来我市在基础设施、新区建设、招商引资等方面所奠定的基础、积蓄的潜能，丹东从未像现在这样有这么多的大项目；信心来自于对未来的热切期盼，要看到我们谋划的宏伟构想正在一步步实现，丹东从未像现在这样满怀激情，向着建设大丹东的目标大踏步迈进。

我们的思路已经清晰，目标已经明确，关键是要抓落实。因此，确定今年的主题仍然为“落实年”，政府工作总的要求是：全面贯彻落实党的十七大、十七届四中全会和中央经济工作会议精神，以邓小平理论和“三个代表”重要思想为指导，深入贯彻落实科学发展观，按照市委十届九次全会提出的“一核两翼两大板块”的部署，在迅速做大做强全市经济总量的基础上，着力加快经济发展方式转变，着力加快以“四大组团”为核心的对外开放步伐，着力加快投资增长，着力加快科技创新，着力加快民生工程建设，高起点开局、高标准落实、高效率运作，力争两到三年实现地区生产总值超千亿、财政一般预算收入过百亿，努力推进丹东的大开发、大开放、大发展。

综合考虑各种因素，2010年全市经济和社会发展主要预期目标是：地区生产总值增长17%，规模以上工业增加值增长23%，财政一般预算收入增长20%，其中各项税收增长25%，全社会固定资产投资增长40%，外贸出口增长10%，实际利用外商直接投资增长40%，引进内资增长50%，社会消费品零售总额增长18%，金融机构新增贷款增长25%，城镇居民人均可支配收入增长12%，农民人均纯收入增长11%，城镇登记失业率、居民消费价格总水平控制在全省平均水平以内。

为实现上述目标，我们必须全力抓好以下重点工作：

（一）以沿江沿海开发开放为牵动，加快对外开放步伐

新区建设有新形象。拓展新区发展空间，新开发土地6平方公里，启动行政中心、安民山公园等城市功能设施建设，加快实施主干路网等基础设施和绿化、亮化工程，确保一批商贸、地产项目开工。港口工业区新拓展3平方公里，保证项目入驻需要。做大仪器仪表产业基地规模，实施核心区三期工程，新建标准厂房80万平方米，完成研发检测中心、物流配送中心、辽宁仪器仪表学院工程，新入驻企业200户。环保产业园启动二期工程建设，累计入驻企业105户以上。促进专业园区项目摆放，确保手表工业园竣工投产，辽表集团及配套企业按时入驻；完善装备制造园区基础设施，推进豪华游艇制造、黄海汽车移地改造等项目建设，引进配套企业；不锈钢产业园依托本钢不锈钢冷轧项目，引进下游企业。

口岸建设有新突破。力争新建鸭绿江公路大桥主体工程开工，加快推进大桥功能区建设，SK保税物流园按期竣工，规划建设2平方公里的保税物流园区。加强大桥建设的宣传力度，提升丹东的国际影响力，使大桥成为牵动丹东发展的新亮点。丹东机场改扩建工程主体完工，积极争取成为国际口岸机场。加快大台子港再生资源码头建设，力争升级为国家一类口岸。

组团开发有新进展。前阳组团加快行政区南移，建设国际小商品城，做大做强服装产业，拉动仓储物流产业发展；东港组团以再生资源产业园为重点，大力发展再生资源、风电装备制造等产业，加快打造临港工业核心区；大孤山组团以海洋红港口建设为牵动，加快城镇建设步伐，推动精密铸造业、旅游业发展。积极推进金山、通远堡、五龙背、九连城等经济区建设。

外资外贸有新增长。以高新技术产业、现代装备制造业、低碳环保产业和新区开发为重点招商领域，以日本、韩国、新加坡和港澳台地区为重点招商区域，强化与世界500强企业等大型跨国公司的联系与合作，力争引进一批具有较强影响力、辐射力的外资大项目。用足用好国家鼓励政策，支持重点出口企业做大做强，推动外贸出口由保份额向保增长转变。

区域合作有新迈进。全力推进“西进东联”，搭建区域合作平台。深化东北东部12市（州）圆桌会议各项工作，积极推进丹通经济带建设；落实大连丹东区域合作框架协议，与大连携手开发北黄海经济带，主动承接大连的产业转移。

（二）以重大项目建设为牵动，形成经济发展新动力

确保项目投资高速增长。全年实施千万元以上项目600　个，其中亿元以上项目194个。力争引进单体投资超百亿元的大项目，投资额在50亿元、10亿元、1亿元以上项目大幅增加。确定重点推进项目80个。

确保项目建设快速推进。竣工一批重点在建项目，确保蒲石河抽水蓄能电站第一台机组并网发电，翁泉沟硼铁综合利用等项目竣工投产，金山热电、丹东（国际）客运站土建主体工程争取完工；推进一批续建项目，丹通和丹海高速公路、东北东部铁路完成路基等工程，本钢不锈钢冷轧等项目完成设备安装；开工一批新建项目，实现沈丹客运专线、丹大快速铁路等项目开工建设；谋划一批前期项目，实现海洋红港口前期工作重大突破，加快谋划和推进东港核电等项目前期工作。丹东港重点建设南1号至3号码头及其他设施，港口吞吐能力突破6000万吨。

确保项目招商实现飞越。以仪器仪表产业基地、新建鸭绿江公路大桥、“四大组团”建设为主题，突出产业园区项目、超百亿元工业项目和战略性新兴产业项目，创新招商体制，完善招商方式，拓展招商领域，精干招商队伍，扩充招商项目库，实现由招商引资向招商选资的转变，促进招商引资再上新台阶。进一步规范招商引资政策，营造有序的招商环境。

（三）以优化产业结构为主线，加快新型工业化进程

积极培育新兴产业。围绕以生物育种等为重点的现代农业，以无水印染设备生产等为重点的先进装备制造业，以高纯度硼粉等为重点的新材料产业，以核电、生物能源等为重点的新能源产业，以新型电子元器件等为重点的电子信息产业，以生物技术等为重点的生物制药产业，以环境保护技术等为重点的环保产业，抓紧规划包装项目，培育建设一批企业，抢占先机，抢先发展。

提升工业经济比重。加快培育发展重点产业、重点园区和重点企业，保持工业经济快速增长。主营业务收入增长35%，税金增长22%。壮大工业经济实力，两年时间内力争实现三个50%的目标，即投资增长速度不低于50%、在固定资产投资中所占比重不低于50%、工业增加值在地区生产总值中所占比重不低于50%。

加快工业园区建设。加快发展以仪器仪表产业基地、环保产业园为重点的新区各工业园区，以再生资源园区等为重点的“四大组团”工业园区，以硼产业基地、黄金产业基地等为重点的县域及城区特色产业工业园区。全年园区新建标准厂房160万平方米。

加大工业投入力度。工业投入360亿元，新开工千万元以上项目150个，其中超亿元项目50个。争取引进更多与央企合作项目。新增规模以上工业企业100户。加速推进高新技术产业发展，新培育省级企业技术研发中心4—5户，进一步加强产学研合作。深化国企改革，积极推进企业并购重组。

推动中小企业发展。强化经济运行，落实各项政策，促进停产企业恢复生产并确保不再停产，抓好企业产品销售，保持企业用电量持续增长。协调金融机构放宽准入条件，积极为劳动密集型中小企业解决贴息贷款。提高担保能力，担保机构数量达到20家，担保贷款总额突破8亿元。

促进节能减排降耗。鼓励发展循环经济，支持清洁生产，倡导低碳生活，万元GDP能耗1.42吨标准煤，下降4.5%；化学需氧量、二氧化硫排放量分别控制在4.7万吨、3.96万吨以内。

（四）以提速升位为着力点，壮大县域经济实力

壮大县域经济促协调发展。三县（市）积极争进全国百强县和全省十强县。规划并组织实施“一县一业”。加速推进农村工业化进程，各县（市）均建成1个以上特色产业园区，并初步形成各自的主导产业。三县（市）按照建设中等城市的要求完成规划的修订和完善，房地产开发面积当年均达到40万平米以上。

发展现代农业促农民增收。以一产实现两位数增长为目标，以农民增收为核心，提高农业综合生产能力，抓好优质稻米、有机果菜等特色产业开发，加快发展现代畜牧业和特色水产养殖业，新增设施农业面积1万亩。大力扶持农业产业化龙头企业，新增规模以上企业30家。健全农产品质量安全监管体系，新增标准化安全农产品生产面积20万亩，新增“三品”质量认证40个。加强和完善科技特派工作，加强对农民的科技培训，大力发展农村专业合作组织。

完善农村基础设施促新农村建设。继续实施鸭绿江界河整治等防洪工程建设。全面完成大中型及重点小型病险水库除险加固任务，加快改造维修农田灌排工程、灌区节水示范项目，通过提水入库等措施解决春灌缺水问题，抓好涝区治理，推进小型农田水利工程建设。做好农村土地开发整理。全面落实惠农政策。加强农村生活污水和垃圾的综合利用和处理，着力解决农村人口饮水安全问题。大力抓好农村沼气国债项目和农村秸秆气化集中供气工程建设。进一步推进“兴边富民行动”。

（五）以提升城区经济实力为突破，促进服务业大发展

发展现代服务业打造城区经济新亮点。抓紧规划建设“一园三区”，即以仪器仪表检测中心为代表的高科技产业园；以新城区金融大厦等为中心的中央商务区；以五龙背、汤池、金山等旅游资源为依托的文化休闲旅游区；以新建鸭绿江公路大桥为核心的现代物流功能区。大力发展以总部经济为重点的现代服务业，着力解决城区经济发展瓶颈，建设现代化城区。抓紧推进沃尔玛超市、东北亚国际商贸城等新建续建服务业重点项目，争取黄海大市场等3家大型农产品批发市场进入国家“双百市场工程”。抓好房地产销售，拓展房地产销售市场。

提升旅游业展现最美丹东新魅力。围绕打造中国最佳旅游目的地城市，以建设鸭绿江精品旅游带开发为核心，抓好虎山景区综合改造、“北国之春”温泉度假村等旅游项目建设，加快鸭绿江景区基础设施建设，创建国家级5A级景区。打造赴朝旅游集散地。做好旅游宣传促销，开拓国内外市场。筹办好鸭绿江国际旅游节和第九届辽宁台湾周活动。

（六）以财政金融支持为重点，增强资金保障能力

提高财政运行质量。以培育财源为基础，提高税收收入和市本级财力比重，涵养财源；以依法组织税收为手段，落实增收挖潜措施，拓宽增收渠道；以民生领域和社会事业支出为重点，优化支出结构，深化财政改革；以加强政府债务管理和国有资产保值增值为途径，落实防范和化解财政风险措施。

增强金融支持能力。实现新增贷款100亿元。积极争取域内外银行基础设施建设贷款及域内银行增加信贷投放，大力支持重大项目、“四大组团”及重点园区的贷款需求。注重引进域外银行机构，积极开办村镇银行和小额贷款公司。确保欣泰电气上市。

（七）以保障和改善民生为根本，推进和谐社会建设

切实做好惠民实事工程。为群众办实事15件：1、机关、企业帮扶贫困村，解决低收入人口生产生活困难；2、实施城市内河治理改造工程；3、改造大修20条城市道路；4、实施三号干线贯通工程；5、打通2条南北道路；6、改造花园路及城市出口；7、实施老城区立面、广告、亮化等综合整治；8、改造城市环卫设施，逐步取消主城区沿街垃圾箱；9、建设文化中心、体育中心；10、开工建设新区医院；11、改扩建市中心医院；12、建设残疾人康复中心；13、建设3万平方米、600套廉租房；14、帮助1000户农村低保户新建维修住房；15、提高烧煤取暖户补贴标准。

不断完善社会保障体系。抓好大中专毕业生和农民工就业工作，加强职业技能培训和公共就业服务，全年实现实名制就业5万人。继续做好养老保险、失业保险、医疗保险扩面工作，加快解决失地农民养老保险问题。新农合人均筹资标准提高到150元，农村常住人口参合率提高到99.3%。全面启动“金保工程”建设。做好低保户、五保户及优抚对象的救助保障。

强化城市规划建设管理。加快推进城市总体规划修编工作。实施一批改善城市环境的重点工程。全面开展市容、交通等专项整治活动，加大对占道经营、私搭乱建等顽疾的清理力度。加快城市东部基础设施建设。强化同城化连接带路网等基础设施建设，加快黄海大道两侧开发，力争开通老城区至新区的水上巴士。制定完善同城化产业发展规划。加快推进各项社会事业的同城化。

积极推进各项事业发展。凤城市、宽甸县、元宝区、振安区通过省“双高”普九验收。完成中小学校舍安全工程阶段性任务，以及市中等职业技术专业学校异地新建工程。完成市新华书店转企改制，启动市专业艺术团体体制改革。实施数字电视双向高清整体转换。全面推进医改五项重点任务。启动第一医院、第三医院改扩建工程。实施基本药物制度。促进公共卫生服务均等化。推动全民健身活动深入开展。落实计划生育各项奖励扶助政策。协调发展老龄事业。积极开展全民国防教育，切实做好双拥工作。认真抓好边海防和民兵预备役建设。

全力打造和谐平安丹东。切实加强社会治安防控体系建设，严密防范、严厉打击境内外敌对势力的破坏活动，有效维护国家安全和边境秩序。切实抓好民族宗教工作。加大打击传销、无照经营工作力度，稳定市场物价，保障食品、药品安全，维护市场经济秩序。努力提高应急管理能力和水平。高度重视信访工作，积极化解各种矛盾。深入开展安全生产专项整治，有效防范和坚决遏制重特大事故发生。

全面提高城乡文明水平。深入开展城市精神宣传活动，不断提高公民思想道德素质和文明素质，特别要加强未成年人思想道德建设。大力加强农村精神文明建设，深化城乡共建、道德信贷活动。深入开展“信用丹东”活动，全力打造诚信丹东。

（八）以服务群众为宗旨，建设人民满意政府

建设勤政高效政府。继续深入开展“落实年”活动，大力发扬“白加黑”、“五加二”工作作风。落实绩效评估工作，加大目标考核和督查力度。打破体制界限，对埋头苦干、脚踏实地的干部积极向党委推荐提拔使用，对作风飘浮、不干实事的坚决按照有关规定进行处罚、调整或撤换。推进市县政府机构改革，强化政府管理创新，建设人民满意的公务员队伍。

建设民主法治政府。坚持依法行政，涉及重大项目实施、重大资金使用等问题，主动向市人大报告、向市政协通报。涉及全市产业布局、发展规划等重大问题，虚心听取专家的意见，做好充分论证，超前谋划“十二五”各项工作。涉及民生的重大问题，坚持行政决策听证制度，自觉接受人民群众通过来信来访、新闻媒体、互联网络等各种途径的监督，提高政府公信力。认真办理人大代表建议和政协提案，提高落实率。

建设清正廉洁政府。严格实行问责制，抓好软环境建设，加强效能监察，对重大项目、重点工程和民生工程实施监督检查。搞好以工程建设领域突出问题专项治理为重点的行政执法监察，深入开展教育乱收费、医药购销等群众反映强烈问题的专项治理。加大对违纪违法行为的查处力度，严厉查办官商勾结、商业贿赂、破坏软环境建设、损害群众利益的重大案件。推进治本抓源，建立和完善政府廉政建设的各项制度。

各位代表，前进的号角催人奋进，跨越的脚步勇往直前。我们坚信，只要在市委的正确领导下，在市人大、市政协的监督和支持下，高举中国特色社会主义伟大旗帜，深入贯彻落实科学发展观，坚定“超千过百”的发展信念，保持持续跨越的良好势头，抓住机遇，咬住目标，满怀激情，乘势而上，就一定能够在新一轮沿海开发开放中，夺取更大的胜利！
